# UNDERSTANDING SOCIAL ISOLATION AMONG MEXICAN AMERICAN ALZHEIMER’S CAREGIVERS: ROLE OF TECHNOLOGY

**DOI:** 10.1093/geroni/igae098.0327

**Published:** 2024-12-31

**Authors:** Lin Jiang

**Affiliations:** University of Texas-Rio Grande Valley, Edinburg, Texas, United States

## Abstract

The projected increase in Latinos affected by Alzheimer’s disease and related dementia (ADRD) to 1.3 million by 2050 underscores the importance of understanding the experiences of Mexican American family caregivers. Despite recognized benefits of information communication technology (ICT) for caregivers, current research predominantly focuses on White American caregivers, leaving a gap in understanding the unique challenges faced by Mexican American caregivers. This study addresses this gap by conducting semi-structured, in-depth interviews with 24 Mexican American family caregivers of ADRD patients along the South Texas and Mexico Border. Participants were recruited through various channels, and Zoom interviews were conducted from January to Fall 2023, followed by thematic analysis. Participants ranged in age from 24 to 73, with an average of a college degree. Fourteen identified as Catholic, while seven identified as Christian. The average caregiving duration ranges from 1 to 13 years. Key findings reveal distinct experiences of social isolation and loneliness, aggravated by a lack of understanding from family members, with ICT playing a significant role in reducing social isolation, particularly during the COVID-19 pandemic. Additionally, religion emerged as a vital coping mechanism, alleviating social isolation and loneliness through online activities. The study highlights disparities in social isolation and emphasizes the potential of technology in addressing these challenges among Mexican American family caregivers of ADRD patients. Recognizing their unique obstacles and leveraging technology can inform interventions to enhance well-being and support. Future research should prioritize culturally sensitive ICT interventions to improve the quality of life for both caregivers and patients.

